# Prospective study of the natural history of chronic acid sphingomyelinase deficiency in children and adults: eleven years of observation

**DOI:** 10.1186/s13023-021-01842-0

**Published:** 2021-05-10

**Authors:** Margaret M. McGovern, Melissa P. Wasserstein, Bruno Bembi, Roberto Giugliani, K. Eugen Mengel, Marie T. Vanier, Qi Zhang, M. Judith Peterschmitt

**Affiliations:** 1 Hsc T-4 Ste 169, Stony Brook University School of Medicine, Stony Brook, NY 11794 USA; 2 Children’s Hospital at Montefiore, Albert Einstein College of Medicine, Bronx, NY USA; 3 Academic Medical Centre Hospital of Udine, Udine, Italy; 4 Med Genet Serv and DR BRASIL Research Group, HCPA, Department of Genetics, UFRGS, and INAGEMP, Porto Alegre, Brazil; 5 Institute of Clinical Science in LSD, SphinCS, Hochheim, Germany; 6 Hôpitaux de Lyon and INSERM, Lyon, France; 7 Sanofi Genzyme, Cambridge, MA USA

**Keywords:** Niemann-Pick disease type B, Niemann-Pick disease type A/B, ASMD, Lysosomal storage disease, Natural history

## Abstract

**Background:**

Acid sphingomyelinase deficiency (ASMD) (also known as Niemann-Pick disease types A and B) is a rare and debilitating lysosomal storage disorder. This prospective, multi-center, multinational longitudinal study aimed to characterize the clinical features of chronic forms of ASMD and disease burden over time in children and adults.

**Results:**

Fifty-nine patients (31 males/28 females) ranging in age from 7 to 64 years with chronic ASMD types A/B and B and at least two disease symptoms participated from 5 countries. Disease characteristics were assessed at baseline, after 1 year, and at the final visit (ranging from 4.5 to 11 years). Thirty patients (51%) were < 18 years at baseline (median age 12 years), and 29 were adults (median age 32 years). Overall, 32/59 patients completed the final visit, 9 died, 9 discontinued, and 9 were lost to follow up. Common clinical characteristics that tended to worsen gradually with time were splenomegaly, hepatomegaly, interstitial lung disease, lung diffusion capacity (DL_CO_), and dyslipidemia. Spleen volumes ranged from 4 to 29 multiples of normal at baseline, and splenomegaly was moderate or severe in 86%, 83%, and 90% of individuals at baseline, year 1, and final visits, respectively. The proportion of all individuals with interstitial lung disease was 66% (39/59) at baseline and 78% (25/32) at the final visit, while median % predicted DL_CO_ decreased by > 10% from baseline to the final visit. Nine patients died (15%), eight of causes related to ASMD (most commonly pneumonia); of these eight patients, five (63%) had symptom onset at or before age 2. Overall, six of the nine deaths occurred before age 50 with three occurring before age 20. Individuals with either severe splenomegaly or prior splenectomy were ten times more likely to have died during the follow-up period than those with smaller or intact spleens (odds ratio 10.29, 95% CI 1.7, 62.7). Most children had growth deficits that persisted into adulthood.

**Conclusions:**

This study provides important information about the natural history of chronic ASMD and provides a longitudinal view of the spectrum of disease manifestations and major morbidities in children and adults and supports the selection of clinically meaningful endpoints in therapeutic trials.

## Introduction

Acid sphingomyelinase deficiency (ASMD) is a rare lysosomal storage disorder that leads to the accumulation of sphingomyelin (and other lipids) in cells and tissues due to deficient acid sphingomyelinase activity (ASM, *SMPD1*; EC 3.1.4.12) [1]. Birth prevalence is estimated to be 0.4–0.6/100,000 [2]. The numerous sequence variants in the *SMPD1* gene and variability in residual ASM activity, as well as other genetic and epigenetic factors, result in a spectrum of ASMD disease severity from a uniformly fatal form with death by 3–4 years of age (infantile neurovisceral ASMD type A, Niemann-Pick Disease [NPD] type A) [3]), to chronic forms characterized by visceral disease [4].

Chronic visceral ASMD (ASMD type B, NPD type B) is characterized by hepatosplenomegaly, thrombocytopenia, interstitial lung disease, and dyslipidemia with potential resultant premature cardiac and vascular disease, in the absence of overt neurological involvement [4]. Bleeding episodes are common in part due to complications from thrombocytopenia and/or to abnormal platelet function. Additional disease manifestations of chronic visceral ASMD may include chronic pulmonary infections, anemia and leukopenia, delayed growth and puberty, osteopenia with increased fracture risk, and progressive liver fibrosis leading to hepatic dysfunction or liver failure. An intermediate form of ASMD, chronic neurovisceral (type A/B ASMD) [5] has neurologic involvement in combination with visceral disease. However, the neurological involvement is less severe than that occurring in infantile neurovisceral ASMD and can include neurocognitive delay, hypotonia, and peripheral neuropathy [4, 5]. Several studies have looked at the ASMD disease burden over time [4, 6–8], and determined that chronic ASMD is associated with stable to slowly progressing pulmonary disease, liver function, thrombocytopenia, and splenomegaly, as well as early mortality. Chronic forms of ASMD (types A/B and B) can be associated with early mortality due to respiratory or liver failure in both pediatric and adult patients [9], with pediatric patients at particular risk for early death [7, 10].

Currently, there is no specific treatment available for ASMD, although initial clinical trials with olipudase alfa enzyme replacement therapy have shown promise [11, 12, 13]. After 30 months of treatment in 5 adult patients with chronic visceral ASMD, olipudase alfa reduced spleen volumes by 50% and improved lung function and infiltrative lung disease pathology [12]. A phase 2/3 randomized placebo-controlled clinical trial in adults (NCT02004691; EudraCT: 2015-000371-26) and a phase 1/2 study in children [13] (NCT02292654; EudraCT: 2014-003198-40) with chronic ASMD are completed, and patients continue receiving olipudase alfa in a long-term extension study (NCT02004704; EudraCT:2013-000051-40).

In this paper, we describe the spectrum of disease manifestations and disease-related morbidity and mortality identified over 11 years of observation in children and adults with chronic visceral or neurovisceral ASMD (types A/B and B), hereafter referred to as chronic ASMD.

## Methods

### Study design and patients

Fifty-nine patients with chronic ASMD were enrolled between May 5, 2001 and June 17, 2002 in a longitudinal study to collect prospective natural history data. Patients were enrolled at five sites in the United States (n = 26), Brazil (n = 13), Italy (n = 8), France (n = 7), and Germany (n = 5). Baseline data collected during a 2–3 day period have been previously reported [14]. Institutional Review Board, Ethics Committee, or Human Subjects Safety Committee at each site approved the study. Written informed consent was obtained from each patient and/or their guardian.

Children at least 6 years of age and adults with diagnosis of chronic ASMD were eligible if deficient ASM activity was demonstrated in peripheral leukocytes or cultured skin fibroblasts/lymphocytes and at least two characteristic clinical features of ASMD were present. Female participants of childbearing age had negative pregnancy tests and were not lactating. Participants were excluded if they had received an investigational drug within 30 days of enrollment, had a co-morbidity or other circumstance that would interfere with study participation or assessments or were affected with infantile neurovisceral ASMD type A.

### Clinical assessments

Medical histories, physical examinations, routine clinical laboratory assessments including biomarker levels, neurologic examinations, and ophthalmologic examination were performed at each visit. Height and weight Z-scores were determined using normative growth data from the Centers for Disease Control (www.cdc.gov/nccdphp/dnpa/growthcharts/sas.htm).

Pulmonary function testing including carbon monoxide diffusing capacity of the lung (DL_CO_) adjusted for hemaglobin (Hb) was performed by standard clinical techniques and results expressed as a percentage of predicted values. Exercise tolerance was assessed by the 6-min walk test (6MWT).

### Imaging studies for organomegaly and interstitial lung disease

Computed tomography scans or magnetic resonance imaging of the abdomen were done to determine liver and spleen volumes. Chest radiograph and high-resolution computed tomography (HRCT) were used for assessment of interstitial lung disease (ILD). All image data were collected centrally by a medical imaging core laboratory and reviewed by a board-certified radiologist blinded to patient and time point who scored the degree of ILD from 0 to 3 where 0 = no ILD, 1 = mild (affecting 1–25% of lung volume), 2-moderate, (affecting 26–50% of lung volume, and 3 = severe (affecting 51–100% of lung volume). Liver and spleen volumes were calculated by integrating cross-sectional images and were expressed as volumes and also as multiples of normal (MN), assuming respective normal spleen and liver volumes of 0.2% and 2.5% of body weight (with 1 kg equivalent to 1 L) [15]. Severity ratings of mild, moderate or severe organomegaly, respectively, were < 1.25 MN, ≥ 1.25 to ≤ 2.5 MN, and > 2.5 MN for liver and < 5 MN, ≥ 5 to ≤ 15 MN, and > 15 MN for spleen [15].

### Genotype

Genotyping was performed upon entry into the study. Genotype nomenclature used in this paper conforms to reference sequence NM 00543.4 (Gen-Bank NG_011780.1).

### Statistical analysis

Continuous variables were summarized using descriptive statistics. Longitudinal analyses with hazard ratios and 95% confidence intervals were conducted to evaluate whether a history of splenectomy and/or spleen volume (MN) or liver volume (MN) were risk factors for death. Children and adult mortality outcomes were analyzed using Kaplan–Meier survival analysis where survival probability was calculated as the number of patients surviving divided by the number at risk.

## Results

### Patient disposition and ASMD history

**Fig. 1:**
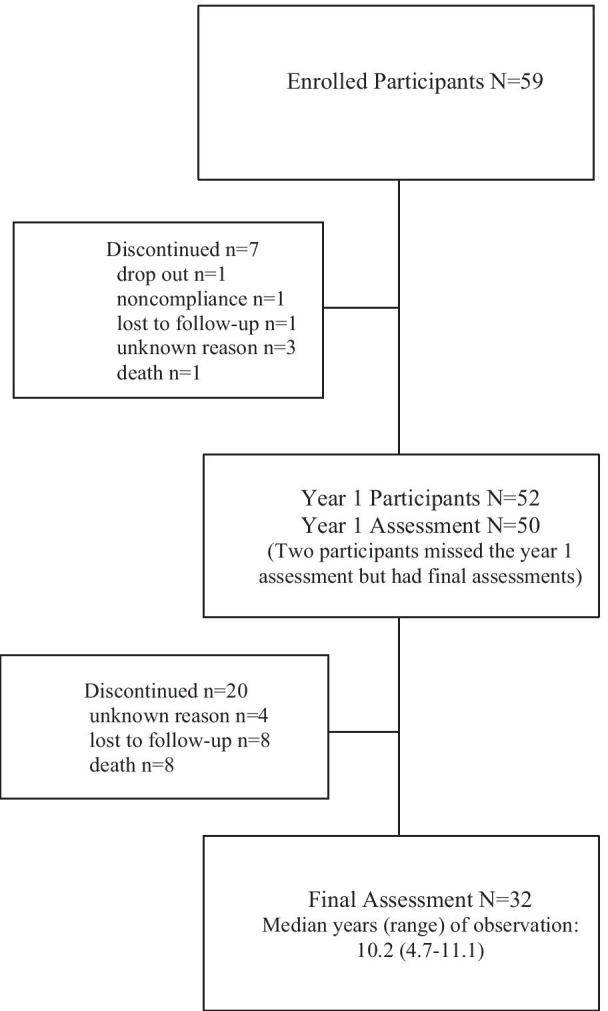
Disposition of study participants

Thirty-one males and 28 females with baseline assessments were enrolled. Patients were predominantly Caucasian (54/59, 92%) and ranged in age from 7 to 64 years (mean 22.0 ± 13.8, median 17.0). Fifty of the 59 participants returned for a 1-year assessment and 32 had a final assessment (Fig. 1). Median years (range) of observation was 10.2 (4.7–11.1).

Patient demographics and disease history stratified by age group at study entry are shown in Table 1. Thirty participants (51%) were in the pediatric age group (< 18 years at start of the study) with a median age of 12 years (range 7–17 years). Among participants in the pediatric group, 12 were under 12 years of age and 18 were between 12 and 17 years at baseline. There were 29 adults (≥ 18 years at start of the study) enrolled with a median age of 32 (range 18–64 years). Given the length of follow up, the proportion of the population that were children changed between the baseline and final assessments (Table 1). At baseline and the year 1 assessment, 49% and 48%, respectively, of individuals were 18 years of age or older, and at the final visit, 97% (31/32) were adults with over half (59%) older than 24 years of age.

**Table 1:** Population characteristics and disease history

Parameter	Pediatric cohort (age < 18 at baseline) N = 30			Adult cohort (age ≥ 18 at baseline) N = 29		
Age range at baseline year	7–17			18–64		
Age distribution n (years)	BL N = 30	Yr1 N = 28	Final N = 15	BL N = 29	Yr1 N = 22	Final N = 17
6–11	12	7	0	0	0	0
12–17	18	19	1	0	0	0
18–23	0	2	10	10	9	2
24–30	0	0	4	3	2	2
31–40	0	0	0	9	7	4
41–50	0	0	0	5	3	7
> 50	0	0	0	2	1	2
Sex, n (%)						
Male	20 (66.7)			11 (37.9)		
Female	10 (33.3)			18 (62.1)		
Ethnicity, n (%)						
Caucasian	27 (90.0)			27 (93.1)		
Black	0			1 (3.4)		
Hispanic	0			0		
Asian	0			0		
Other	3 (10.0)			1 (3.4)		
Age at symptom onset						
Mean (SD), years	2.52 (2.8)			7.94 (8.8)		
Median (range)	2.00 (0.1,14.0)			4.00 (0.9, 30)		
Age at diagnosis						
Mean (SD), years	5.23 (3.6)			15.10 (13.5)		
Median (range)	3.63 (1, 14.3)			11.00 (2, 45.1)		
Splenectomy n (%)						
Partial	0			1 ( 3.4)		
Total	0			4 ( 13.8)		

Mean age at first symptom onset occurred earlier in the pediatric group than the adult group: 2.5 ± 2.8 (range 0.1–14.0) years versus 7.9 ± 8.8 (range 0.9–30) years. The mean age at diagnosis was also younger in the pediatric group (5.2 ± 3.6 years, range 1−14.3) compared to the adult group (15.1 ± 13.5 years, range 2−45.1).

Details of presenting and historical signs and symptoms have been previously described for this cohort (see Table 1 of reference [14]). The majority of participants presented with normal mental status, cranial nerve function, sensation, muscle strength, and coordination. Fifteen of the 59 (25%) had bilateral cherry red macula, five of whom (33%) were cognitively impaired. Most patients presented with splenomegaly or hepatomegaly. Bleeding, shortness of breath, pulmonary infections, and joint/limb pain were frequent historical complaints [14].

## Biomarkers

Chitotriosidase levels were approximately 5–6 times higher than the upper limit of normal at all time points (approximately 500–600 µmol/L/h at baseline, year 1, and year 10, and 1815 µmol/L/h at year 11 [reference range, 0.6–98.4 µmol/L/h]). The mean plasma sphingomyelin levels at all time points were between 221 and 446 µmol/L (normal range, 21.49–412.8 µmol/L). The mean leukocyte sphingomyelin level, obtained only at baseline, was relatively higher at 77 nmol/mg protein (reference range, 25.3–46.2 nmol/mg protein), and 63% of patients had elevated sphingomyelin levels in leukocytes. The mean sphingomyelin level in dried blood spot (DBS), obtained only at years 10 and 11, was 950 µg/mL and 1002 µg/mL, respectively (reference range: 1020–1495 µg/mL).

Analyses of correlations of biomarkers and other clinical parameters with organ volume/disease severity resulted in large data variability with wide confidence intervals, low coefficients of determination, and p values that were greater than 0.05, so no conclusions could be drawn.

### Genotype

Two mutant alleles in the *SMPD1* gene were identified in all participants, representing 48 genetic variants, most of them already described [16–18]. Missense mutations were the most common variants (59% of alleles), followed by deletion of arginine at codon 610 in exon 6 (25%), frameshift (8%) and nonsense (8%) variants. The most common individual variant was the deletion of arginine at codon 610 in exon 6 (p.R610del) which accounted for 25% of alleles (homozygous in 4 individuals and compound heterozygous in 21). When heterozygous, the p.R610del allele was associated with either a missense (n = 15), a stop/nonsense (n = 5), or a frameshift (n = 1) mutation. The second most frequent variant was p.R476W (9 alleles, 7.6%). Four individuals carried one severe p.R498L allele [19] in combination with p.R610del (n = 2) or p.R476W (n = 2). Two individuals were homozygous for the apparently null mutations p.M1T (now M1_W32del) or p.W32* [18, 20].

Two individuals were homozygous for the p.Q294K variant that has been associated with the chronic neurovisceral phenotype type A/B [21], and both had cherry red macula and cognitive impairment at baseline and last follow-up. One individual entered the study when 14-year old, had cognitive impairment with spatial deficit and dyscalculia, normal coordination, and died from pneumonia at age 20 (Table 2). The other was 13-year old at baseline, and 23 at last visit. They had mental retardation with a slow speech that became inarticulate, a wadding gate that progressed to ataxia, coordination problems, and peripheral neuropathy. Three other individuals had cognitive impairment and cherry red macula. The individual with a p.L105P/p.T544fs*69 genotype (Table 2) had severe cognitive impairment at baseline, and died from visceral complications 2 years later, at the age of 17 years. One individual was homozygous for p.L139P (a common variant described as correlating to a mild ASMD type B phenotype [17]) and showed psychomental delay (2–4 years below age at the age of 15 years), without apparent progression and normal neurological exam at all visits. Another individual with a p.W393G/ p.R498C genotype had slight dysarthria at 13 yars of age, rather than a cognitive impairment, and showed ataxia and coordination problems 10 years later. The p.W393G variant has been reported to give varying clinical pictures [22], and no correlation is known for p.R498C. A sixth individual with a p.R443X/p.P325A genotype had cognitive impairment without a record of cherry red macula and had long standing learning disabilities and did not finish 12th grade (age 18 years at baseline and 23 years at last visit). They had a partial splenectomy at age 11. The p.P325A variant is associated with milder disease when homozygous [4] while R443X has been associated with ASMD type A when homozygous [23].

**Table 2:** Mortality

Cause of death	Genotype variant* (classification)	Severity of splenomegaly at baseline	Age at ASMD onset (years)	Age group at baseline	Age at death (years)
Splenic vein tear	p.C433R/p.A198P (missense/missense)	Severe	1	Pediatric	14
Heart failure, liver failure	p.R610del/p.G247S (deletion/missense)	Severe	1	Pediatric	16
Diarrhea, fever/portal hypertension with esophageal varices	p.L105P/p.T544fs*69 (missense/frame shift)	NA	0	Pediatric	17
Pneumonia	p.Q294K/p.Q294K (missense/missense)	Severe	2	Pediatric	20
Pneumonia	p.L434P/p.L434P (missense/missense)	sx	1	Adult	35
Multiple organ failure/infectious disease	p.F482L/p.F482L (missense/missense)	sx	30	Adult	56
Liver cancer	p.A198P/p.A198P (missense/missense)	Severe	9	Adult	65
Pneumonia	p.L37Wfs*42/p.P186L (missense/frameshift)	sx	8	Adult	71
Death from a gun shot	p.R610del/p.R476W (deletion/missense)	Moderate	1	Pediatric	22

NA, not available; sx, splenectomy

*Reference sequence NM 00543.4 nomenclature

Splenomegaly: moderate ≥ 5 to < 15, severe ≥ 15 MN

Among 17 individuals who dropped out of the study or were lost to follow-up, three were homozygous for p.R610del, and 7 were heterozygous. Two individuals who dropped out had alleles associated with severe phenotypes (p.R498L and p.G247S), one in combination with the p.R476W allele associated with mild disease. Another individual lost to follow-up was homozygous for p.M1T.

### Mortality

**Fig. 2:**
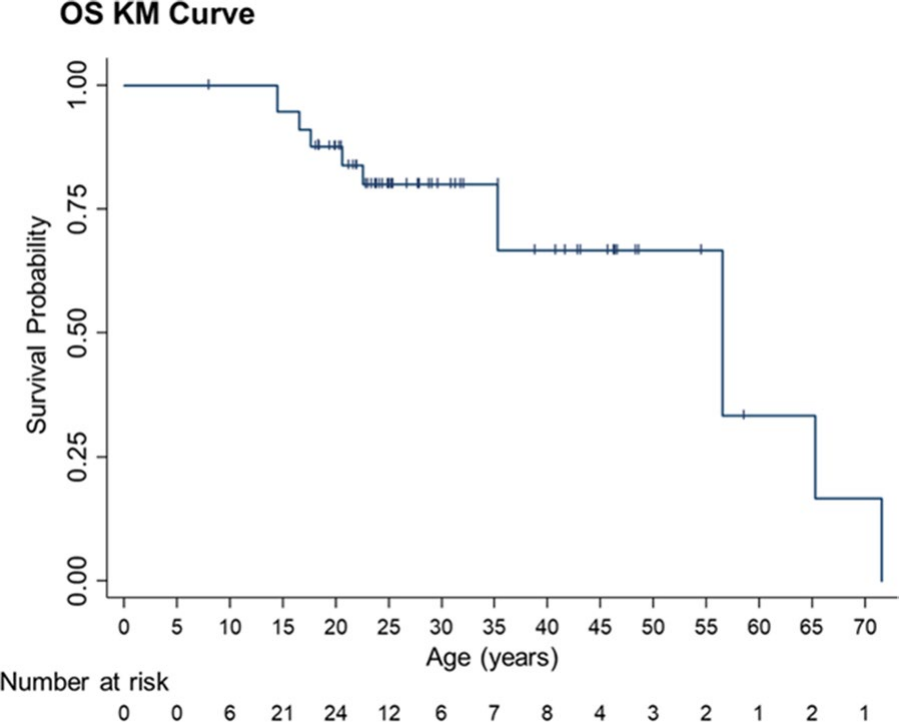
Kaplan–Meier overall survival probability in children and adults with ASMD. The analysis included one patient death unrelated to ASMD (see Table 2)

Nine participants died during the study and causes of death are shown in Table 2. Eight of the nine died from causes associated with ASMD morbidities including lung and liver disease, while one individual died from causes unrelated to ASMD. Death from pneumonia was the most common cause of death (n=3). Figure 2 shows a Kaplan–Meier survival probability curve in children and adult patients with ASMD, where patients who died, dropped out, or not reached the time yet are not counted as at risk (note, there were no patients younger than 7 years of age so there are no patients at risk at 0 and 5 years). Six individuals died before 50 years of age, with three deaths occurring before the age of 20. Early disease onset with symptoms by or at two years of age occurred in six individuals who died (Table 2).

Among those who died from disease-related causes, one individual was heterozygous for the p.R610del variant in combination with the missense variant p.G247S, which is associated with more severe disease [17]. Another individual was homozygous for the p.Q294K variant associated with the chronic neurovisceral type A/B phenotype [21]. Genotype/phenotype correlations are not known for mutations observed in the remaining individuals who died from disease-related causes. The person who died from a non-disease related cause was heterozygous for the p.R610del variant in combination with p.R476W, which is associated with milder disease.

Both total splenectomy and spleen volume 15 MN or greater at baseline appeared to be predictors of mortality. Those with history of either severe splenomegaly or prior splenectomy were 10 times more likely to have died during the follow up period than those with moderate splenomegaly or intact spleens (odds ratio 10.29, 95% CI 1.7, 62.7). Similarly, history of severe splenomegaly alone was associated with increased mortality (odds ratio 6.0, 95% CI 0.81, 44.4). Liver volume of 2.5 MN or more was not a predictor of mortality.

### Pulmonary assessments

The percent of individuals with ILD and reticulonodular density were 66% (39/59) at baseline, 62% (31/50) at 1 year, and 78% (25/32) at the final visit (Table 3). The percentage of individuals with severe ILD was 42% (25/59) at baseline and 50% (16/32) at the final visit. Patients with severe or moderate ILD tended to be younger than those with absent or mild disease (data not shown). More individuals with dyspnea at baseline had moderate or severe ILD at the final visit (13/16, 81%) compared to those without baseline dyspnea (6/16, 38%). Values for the percent Hb-adjusted DL_CO_ tended to decrease (greater impairment) as ILD severity increased (data not shown).

**Table 3:** Proportion of individuals with interstitial lung disease and reticulonodular density determined by high resolution computed tomography

Parameter	Baseline visit	Year 1 visit	Final visit
	(N = 59)	(N = 50)	(N = 32)
Interstitial lung disease		n (%)	
Absent	14 (23.7)	11 (22.0)	3 (9.4)
Present	39 (66.1)	31 (62.0)	25 (78.1)
Mild	10 (16.9)	5 (10.0)	6 (18.8)
Moderate	4 (6.8)	7 (14.0) left; 6 (12.0) right	3 (9.4)
Severe	25 (42.4)	19 (38.0) left; 20 (40.0) right	16 (50.0)
Reticulonodular density		n (%)	
Absent	14 (23.7)	11 (22.0)	3 (9.4)
Present	39 (66.1)	31 (62.0)	25 (78.1)
Mild	10 (16.9)	5 (10.0)	6 (18.8)
Moderate	4 (6.8)	7(14.0) left; 6 (12.0) right	5 (15.6)
Severe	25 (42.4)	19(38.0) left; 20 (40.0) right	14 (43.8)

All values were the same in both lungs except where values for both the right and left lungs are provided

**Fig. 3:**
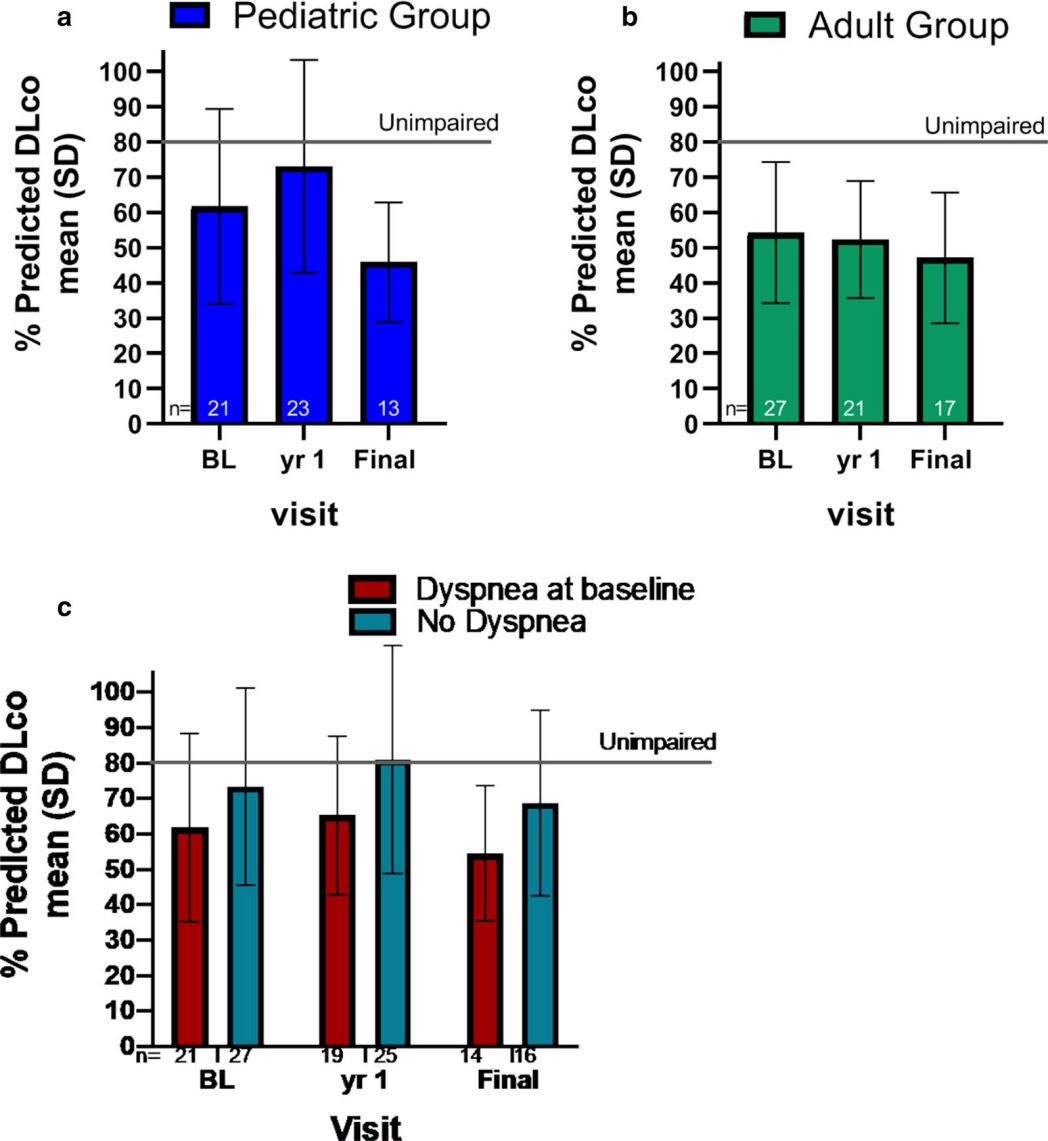
Lung diffusion capacity during the study observation period. Percent predicted diffusion capacity of carbon monoxide (DL_CO_) adjusted for hemoglobin in the pediatric (**a**) and adult (**b**) cohorts at baseline (BL), year 1, and final assessment. Diffusion capacity stratified by presence or absence of dyspnea at baseline for pediatric and adult groups combined is shown in C. Solid horizontal line indicates threshold for abnormal values (< 80% of the predicted normal values)

The mean % predicted Hb-adjusted DL_CO_ for the pediatric and adult groups during the observation period is shown in Fig. 3a, b. The median percent decreases from baseline to the final visit was 12.5% (range − 55 to + 141) in the pediatric group, and 10.4% (range − 44 to + 22) in the adult group. Changes in the mean % predicted DL_CO_ over time and changes from baseline were similar in all patients who had data at all three study visits (data not shown). The population of individuals with dyspnea at baseline had lower mean % predicted DL_CO_ values compared to no dyspnea at baseline (61.8 ± 26.6 vs. 73.4 ± 27.8), and this difference was maintained at each time point (Fig. 3c).

### Cardiopulmonary evaluation

At the baseline visit all individuals were ambulatory, although two required supplemental oxygen, and the mean 6MWT distance was 508 ± 97 m. Three individuals with history of dyspnea had mean 6MWT distances below the 310 m lower limit of normal at the baseline or subsequent visits. One 20-year-old individual was functionally impaired at the final visit (year 11) with 52 m walked, percent predicted Hb-adjusted DL_CO_ of 39.5% and percent predicted FVC of 61%.

### Spleen volume

The majority of individuals had intact spleens (54/59, 92%). Mean baseline spleen volume in non-splenectomized patients was 12.1 ± 5.9 MN (range 3.6–27.2 MN) (n = 42) and splenomegaly ranged from mild (< 5 MN) to moderate (≥ 5 and ≤ 15) to severe (> 15 MN). At the baseline, year 1 and final visit respectively, 86% (36/42) 83% (29/35), and 90% (27/30) of individuals with available data had moderate or severe splenomegaly (≥ 5 MN).

**Fig. 4:**
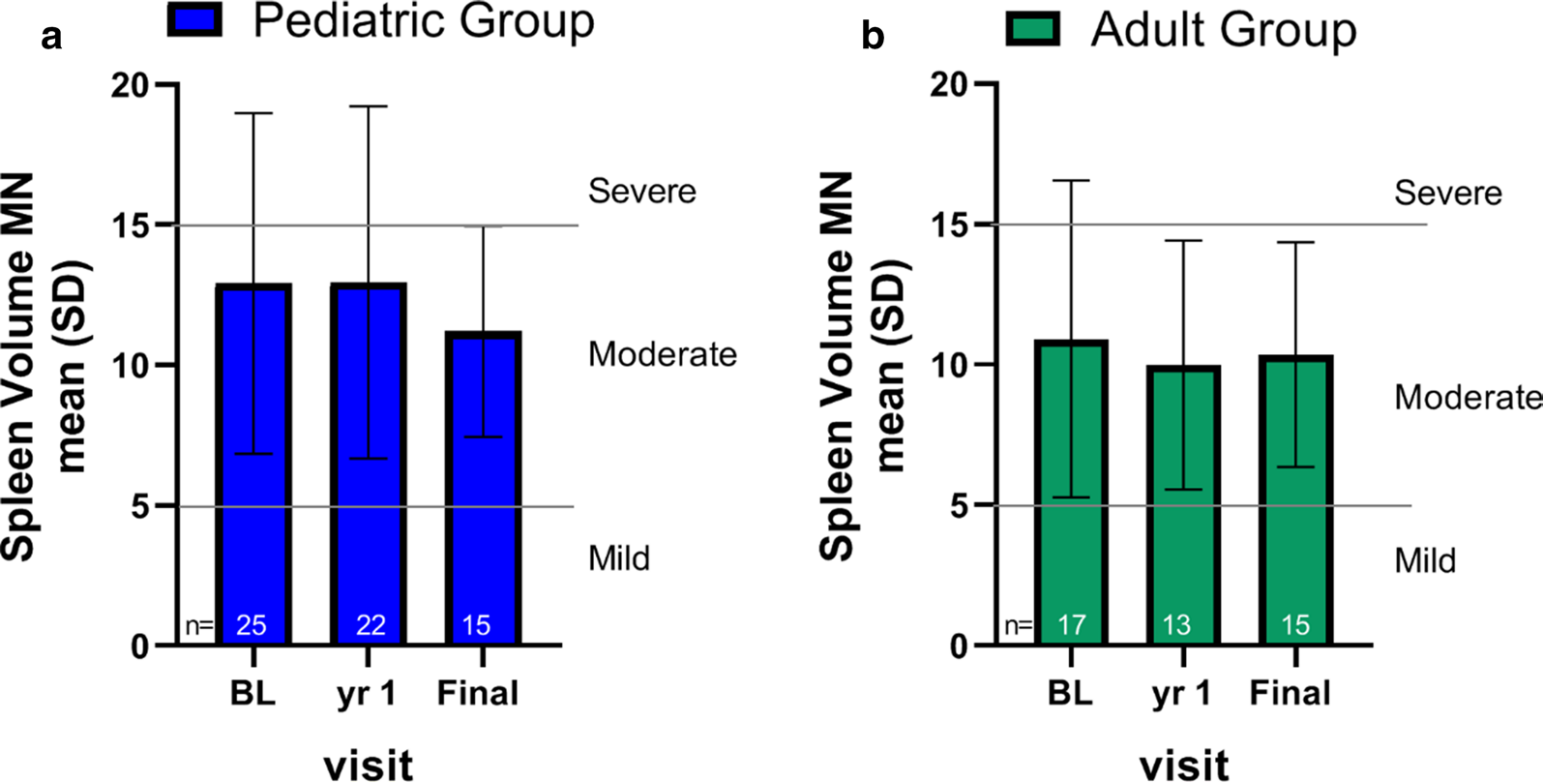
Splenomegaly during the observation period. Spleen volumes in Multiples of Normal (MN) for the pediatric (**a**) and adult (**b**) cohorts at baseline (BL), year 1, and final assessment. Severity of splenomegaly cutoffs are indicated by solid lines where mild is < 5 MN, moderate is ≥ 5 and ≤ 15 MN, and severe is > 15 MN [15]

At baseline, mean spleen volume expressed as MN in the pediatric group was 12.9 ± 6.1 and ranged from 4 to 27 MN. Mean spleen volume in MN over time is shown in Fig. 4a. At the 1 year and final time points, spleen volumes remained stable at 12.9 ± 6.3 and 11.2 ± 3.8 MN, respectively. Of note, approximately half of those who entered the study in the pediatric age group had an increase in spleen volume expressed as MN (increases ranging from 2 to 15%) between the baseline and first year visit, while half had no change or decreases of 0–19%. Those who had declining MN values had substantial changes in their weight and height compared to baseline. Absolute spleen and liver volume (in cm^3^) increased with age in all patients who enrolled as children.

The mean spleen volume expressed as MN at baseline in the adult group was 10.9 ± 5.6 (range 4–26 MN). Mean spleen volume in MN over time remained stable and is shown in Fig. 4b (10.0 ± 4.4 at year 1, and 10.9 ± 2.8 at the final visit).

An analysis of spleen volume over time for the subset of patients with data at all three time points yielded similar results (data not shown).

### Liver volume

**Fig. 5:**
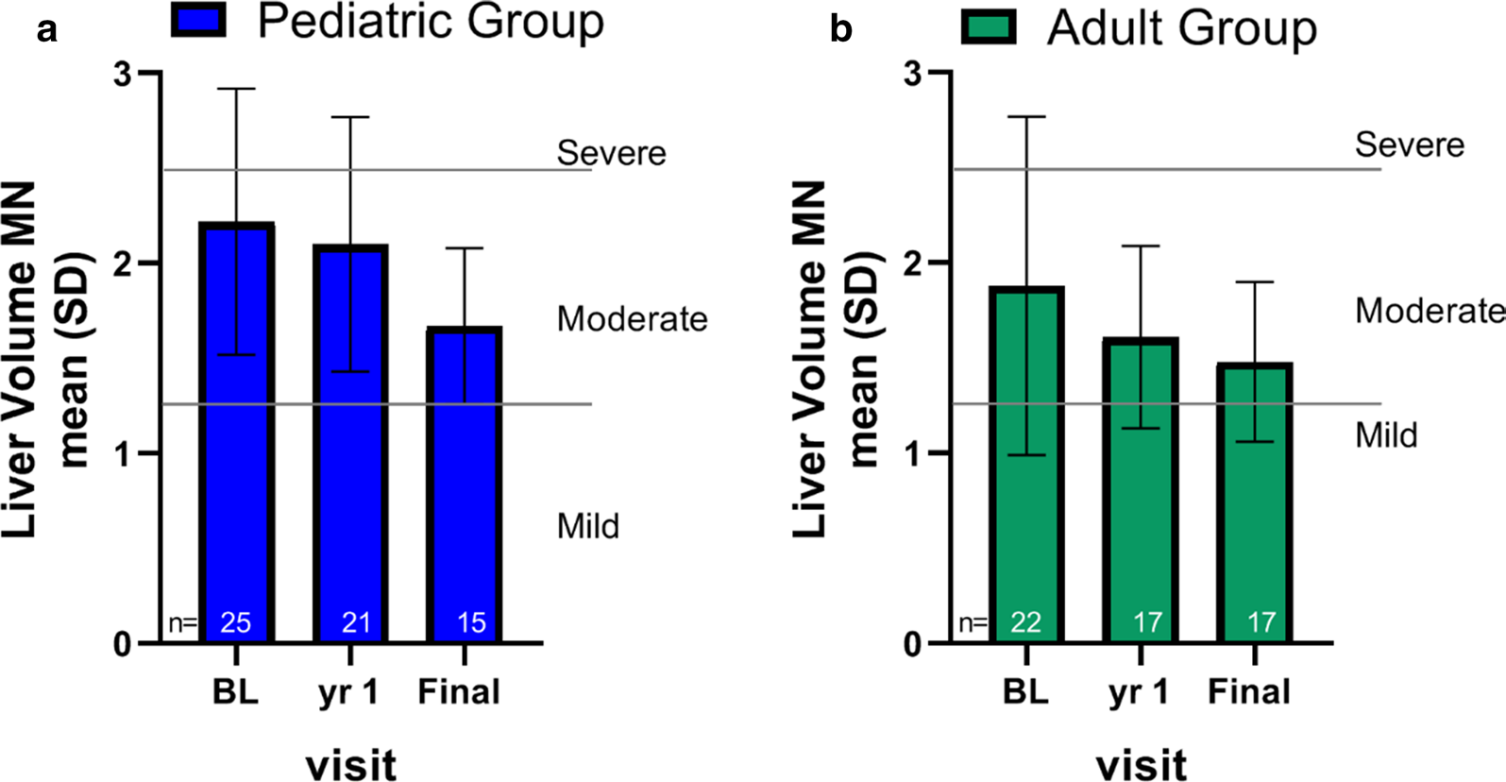
Hepatomegaly during the observation period. Liver volumes in multiples of normal (MN) in the pediatric (**a**) and adult (**b**) cohorts at baseline (BL), year 1, and final assessment. Severity of hepatomegaly cutoffs are indicated by solid lines where mild is < 1.25 MN, moderate is ≥ 1.25 and ≤ 2.5 MN, and severe is > 2.5 MN [15]

Mean baseline liver volume was 2.1 ± 0.8 MN (range from: 0.9–4.6 MN) (Fig. 5a, b for pediatric and adult patients, respectively). Mean liver volume in the pediatric group was 2.2 ± 0.7 MN at baseline and did not change appreciably over time (2.1 ± 0.7 at year 1 and 1.7 ± 0.4 at final visit). In adults, the baseline liver volume of 1.9 ± 0.9 tended to decrease over time to 1.6 ± 0.5 at year 1 and 1.5 ± 0.4 at final visit. As with spleen volume, there was inter-individual variability in the children and adults, with some individuals showing increases, and some showing small decreases in liver volume over time. However, moderate to severe hepatomegaly (1.25–1.75 MN) was observed in the majority of individuals at all time points: 45/47 (96%) individuals at baseline, 37/38 (97%) at year 1 and 28/32 (88%) at the final visit.

An analysis of liver volume over time for the subset of patients with data at all three time points yielded similar results (data not shown).

### Liver function

At baseline, alanine aminotransferase (ALT) and aspartate aminotransferase (AST) were elevated in 47% and 51% of individuals, respectively, while total bilirubin was elevated in 33%. Mean baseline ALT (U/L) was 86.2 ± 67.8 and 51.5 ± 44.6 in the pediatric and adult groups, respectively. Mean ALT values at the final visit were in 65.5 U/L in the pediatric group and 52.7 U/L in adults. Results were similar for AST (data not shown). Total bilirubin was similar at baseline in the adult and pediatric groups (17.5 vs. 21.8 mg/dL, respectively) and values did not change over the observation period. Tests of synthetic function in the liver (including prothrombin time/international normalized ratio, platelet count and albumin level) remained stable or worsened (e.g., thrombocytopenia) during the study.

History of portal hypertension and esophageal varices were described in two individuals, one of whom developed liver failure and died.

### Dyslipidemia

Lipid profiles and changes from baseline over time are shown in Table 4 by age group. Both children and adults had atherogenic lipid profiles at all study visits characterized by high total cholesterol level, low high-density lipoprotein (HDL), high triglyceride level, high low-density lipoprotein (LDL), and high very-low-density lipoprotein levels compared with age- and gender-matched control subjects. Lipoprotein risk ratios of total cholesterol to HDL levels remained elevated during the observation periods in both age groups indicating increased heart disease risk. Statins (HMG-CoA reductase inhibitors) were listed as concomitant medications for three individuals at baseline, two at the year 1 assessment, and two at the final assessment.

**Table 4:** Lipid profiles in the pediatric and adult groups

	Pediatric group (< 18 years at baseline) N = 30			Adult group (≥ 18 years at baseline) N = 29		
	n Mean (SD)					
	Baseline	Year 1	Final	Baseline	Year 1	Final
Total cholesterol (mmol/L)	29 5.71 (1.46)	28 5.46 (1.48)	15 5.35 (1.15)	29 6.08 (2.19)	22 6.19 (2.3)	17 5.63 (1.89)
LDL (mmol/L)	29 4.05 (1.32)	28 3.89 (1.28)	15 3.62 (1.14)	28 4.27 (1.55)	22 4.43 (2.04)	17 4.01 (1.75)
HDL (mmol/L)	29 0.65 (0.28)	28 0.62 (0.27)	15 0.64 (0.28)	29 0.69 (0.25)	22 0.66 (0.25)	17 0.68 (0.35)
TG (mmol/L)	29 2.28 (1.02)	28 2.13 (1.04)	15 2.39 (0.98)	29 2.26 (1.21)	22 2.50 (1.17)	17 2.04 (0.99)
Cholesterol risk ratio (total cholesterol/HDL)	8.8	8.8	8.4	8.8	9.4	8.3

HDL, high density lipoprotein; LDL, low density lipoprotein; TG, triglyceride

*Normal ranges (adults)*: Total cholesterol: US < 5.18 mmol/L; UK 0–3.9 mmol/L; HDL normal range: US male > 0.78; US female > 0.91 mmol/L; UK > 1.2 mmol/L; LDL normal range: US < 3.34 mmol/L; UK 0–2 mmol/L; triglycerides normal range: < 1.7 mmol/L

*Normal ranges (child and adolescent)*: total cholesterol: not available; HDL normal range: 0.78–1.94 mmol/L; LDL normal range: 1.69–3.63 mmol/L; triglycerides normal range: 0.34–1.67 mmol/L

### Hematology

Hematologic abnormalities were mild to moderate in severity at baseline (see Table 2 of reference [14]), and anemia (26% of patients) and leukopenia (21% of patients) reported at baseline did not significantly change over time in either children or adults (data not shown). Platelet counts decreased from baseline to the final visit in both the pediatric and adult groups (mean change from baseline was − 41% and − 12% in the pediatric and adult groups, respectively).

### Growth

**Fig. 6:**
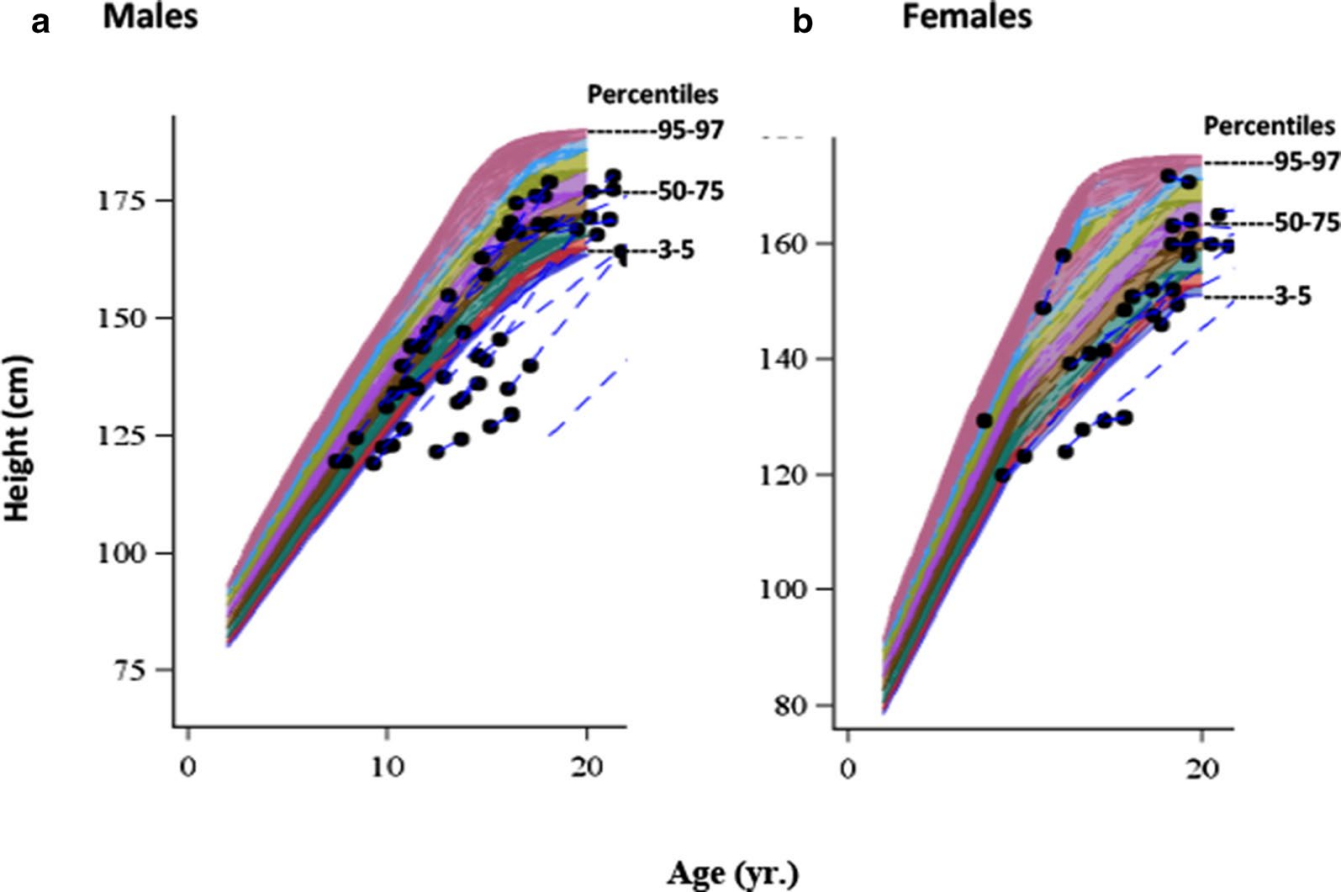
Compromised growth during the observation period. Individual height (cm) by age for males (**a**) and females (**b**) in the pediatric cohort overlaid on CDC growth chart percentiles indicated by different colors with 95–97, 50–75 and 3–5 percentiles marked. CDC normative growth charts stop at age 18. Since growth in some patients continued to be below normative curves after reaching adulthood, data through age 20 are included. Individual patient data are indicated by circles connected by blue dashed lines

For all male and all but three female children, heights and weights were at or below the CDC 50th percentile for age (Fig. 6). In general, final Z-scores in individuals who were children and adolescents at baseline but adults at the final assessment (29/30) remained below normal. Mean height-for-age Z-scores stratified by pediatric age groups are shown in Table 5 and were below − 0.9 at both baseline and the year 1 assessment in children age 6–11 years, and below − 2 in children 12–17 years of age.

**Table 5:** Height and weight in pediatric cohort over time

	Pediatric group Age 6–11 years			Pediatric group Age 12–17 years		
	n Mean (SD)					
	Baseline	Year 1	Final	Baseline	Year 1	Final
Height-for-age Z-Score	12 − 0.9 (1.1)	10 − 1.1 (1.1)	3 − 0.1 (0.6)	18 − 2.7 (1.5)	18 − 2.6 (1.6)	1 − 1.1
Weight-for-age Z-score	12 − 0.7 (0.8)	10 − 0.8 (0.9)	3 0.1 (0.6)	18 − 2.1 (1.7)	18 − 2.2 (2.0)	1 − 1.1

Weights were variable for both male and female children, but most were at or below the fiftieth percentile for age and tended to normalize after age 20 years (data not shown). Mean weight-for-age Z-scores are shown in Table 5 and trends for the two age groups were similar to those for height.

## Discussion

In this prospective study, clinical data over a period of up to 11 years were systematically collected on the largest series of pediatric and adult patients with chronic ASMD reported to date. The data from this series augment previously obtained longitudinal data that documented worsening of pulmonary function, decreasing platelet and white blood cell counts, severe organomegaly, increased liver transaminases, and dyslipidemia [4, 6–8]. In this cohort, below normal growth at baseline continued through the observation period with growth Z-scores for children and adolescents remaining below normal. Previous analysis showed that the majority of adolescent patients had delayed bone ages at baseline indicative of delayed puberty [14]. Individuals in this study showed increased risk of early mortality, with both severe splenomegaly and splenectomy found to be a risk factor for death.

Radiographic evidence for ILD was present in the majority of individuals as has been observed in this cohort and in other studies [24, 25], and increased in severity over time. ILD in ASMD is due to progressive accumulation of sphingomyelin in alveolar macrophages in the interalveolar septae, which impairs oxygen/carbon dioxide exchange [4, 6, 8, 22–24]. As measured by DL_CO_, both children and adults had compromised lung diffusion capacity that worsened over time. Notably, impaired diffusion capacity was most severe in patients with dyspnea, and in those with more severe ILD.

Pulmonary disease in chronic ASMD is associated with increased risk of respiratory infections, which can lead to respiratory failure [9]. In this study, three patients died from pneumonia, two of whom were splenectomized, and another who had severe splenomegaly. In addition, recurrent respiratory tract infections [26] and progressive decline in pulmonary function contribute to decreased quality of life [6, 7, 27–29]. In some cases, pulmonary disease has been the clinical manifestation that drives diagnosis of ASMD. For example, chronic visceral ASMD went undiagnosed until age 52 and 61 years in two adult siblings until progressive dyspnea (with ILD on imaging) prompted the clinical workup and resulting diagnosis [30].

Hepatosplenomegaly is a prominent, early feature of chronic ASMD, with moderate to severe organomegaly observed in most patients. The results of this long-term observational study showed that organomegaly persists, although there was inter-individual variability in organ volumes over time. In order to assess organomegaly in ASMD, and other diseases such as Gaucher, spleen and liver volume are usually expressed as multiples of normal based on calculations that assume normal spleen volume is 0.2% of body weight and normal liver volume is 2.5% of body weight [15, 31, 32]. In healthy individuals, in addition to a high correlation with body weight, spleen size is also highly correlated with height and body surface area in children [33, 34], and height and sex in adults [35]. Therefore, in serial long-term assessments of pediatric patients with ASMD such as this 11-year analysis, during which almost all patients who were children at baseline became adults by their final assessment, multiple body parameters may factor into variation in spleen size relative to body size over time. Despite variability, almost all individuals maintained moderate or severe organomegaly from baseline to the final visit.

Of the nine individuals who died during the study, eight died from ASMD-related causes. Five of these individuals had symptom onset by or at 2 years of age, suggesting that early symptom onset is associated with more severe disease and worse outcomes. As reported in other studies, respiratory infection/failure and liver failure were the most common causes of death [7, 9]. Severe splenomegaly or prior splenectomy were associated with a tenfold increased risk of death compared to individuals with smaller or intact spleens. One individual who died was homozygous for the p.Q294K variant linked to the type A/B chronic neurovisceral phenotype (type A/B), which is associated with greater morbidity than the chronic visceral phenotype (type B) [21]. While most participants in this cohort did not exhibit neurological abnormalities, six patients had cognitive impairment, five of whom had cherry red macula, including both individuals homozygous for the p.Q294K variant, indicative of patients with chronic neurovisceral ASMD type A/B. Two individuals with cherry red macula and cognitive impairment were among those who died during the study: one individual homozygous for the p.Q294K variant who died before the age of 25 from pneumonia, and another with a p.L105P/p.T544fs*69 genotype and severe cognitive impairment at baseline, who died from visceral complications at the age of 17 years. In general, organomegaly and pulmonary function status over time for patients with cognitive impairment were similar to those for individuals without cognitive impairment or cherry red macula.

The p.R610del variant, which is associated with milder disease manifestations [19, 36] was the most common variant found in the study participants, accounting for 25% of alleles. Two adults were homozygous for the apparently null mutations p.M1T (now M1_W32del) or p.W32*, respectively [18, 20]. One had a mild clinical picture at baseline that was stable over the 10 years of follow-up, with normal DL_CO_, mild hepatomegaly, moderate splenomegaly, and mild-to moderate ILD. The other individual was lost to follow-up and had insufficient baseline data to draw any conclusion on disease severity. The mutation p.M1T inactivates the first in-frame initiation codon (ATG) of *SMPD1*. A second ATG has been identified at position 33 and shown to be functional in vitro. Patients homozygous for p.W32* or p.M1T were shown to present with mild ASMD with 20–25% of residual enzyme activity with respect to normal values [16]. Although expression studies resulted in no activity for p.W32*[20], the chronic ASMD clinical phenotype strongly suggests at least some in vivo functionality of the second ATG.

Dyslipidemia has been reported in other cohorts of ASMD patients [8, 37], and may be associated with increased risk of atherosclerosis [34], although additional research is needed to fully understand the impact of dyslipidemia in patients with chronic ASMD. In both children and adults in the current series, lipid profiles were highly atherogenic with mean total cholesterol/HDL ratios > 8, which is associated with increased risk of coronary artery disease. Lipid profiles worsened between baseline and year 1 in both children and adults. Only two individuals were on statin therapy at the year 1 and final assessments, suggesting an unrecognized need in this patient population for lipid management. As previously reported for this cohort, [14] splenectomy was associated with atherogenic lipid profiles, which suggests that redistribution of sphingomyelin to other organs following the removal of a major depot organ can lead to secondary disease acceleration. Future efforts to obtain longitudinal data on cardiovascular disease assessments would be useful for fully understanding the extent of cardiac disease in chronic ASMD.

The results of this study provide further evidence of the progressive disease manifestations in chronic forms of ASMD. A strength of the study is that the cohort included patients from five countries on three continents (North America, South America and Europe). Selection of patients with at least two clinical features of ASMD may have limited enrollment of patients with very mild disease. The challenges of conducting the study include that 9/59 patients (15%) died during the study, and the eight that died of ASMD-related causes were presumably the most severely affected patients. The loss of these patients from the cohort may have led to an underestimation of disease severity. In addition, although 50 of the 59 patients returned for the year 1 assessment only 32 had a final evaluation. It is possible that some of those who were lost to follow-up also had died, while some who dropped out may have had very mild disease and were not motivated to undergo the study evaluations, or conversely, had severe disease and were too ill to continue to participate.

## Conclusions

The results of this study provide important new information about the natural history of disease manifestations in chronic ASMD. The clinical information collected for this large series of children and adults with ASMD worldwide provides a longitudinal view of the spectrum of disease manifestations and major morbidities and supports the selection of clinically meaningful endpoints in therapeutic trials.

## Data Availability

Datasets used and/or analyzed for the study are available upon reasonable request.
